# Epilepsy surgery: Recommendations for India

**DOI:** 10.4103/0972-2327.64625

**Published:** 2010

**Authors:** P. Sarat Chandra, Manjari Tripathi

**Affiliations:** Department of Neurosurgery New Delhi-110 029, India; 1 Neurology, AIIMS, New Delhi-110 029, India

**Keywords:** Epilepsy surgery, hemispherotomy, lesionectomy, Level I, Level II, multiple subpial transactions, resective surgery

## Abstract

The following article recommends guidelines for epilepsy surgery for India. This article reviews the indications, the various surgical options available and the outcome of surgery for drug resistant epilepsy based on current evidence. Epilepsy surgery is a well-established option for patients who have been diagnosed to have drug resistant epilepsy (DRE) (on at least two appropriate, adequate anti-epileptic drugs (AEDs) (either in monotherapy or in combination) with continuing seizures), where the presurgical work-up has shown concordance of structural imaging (magnetic resonance imaging) and electrical mapping data (electroencephalography (EEG), video EEG). There may be a requirement of functional imaging techniques in a certain number of DRE like positron emission tomography (PET), single photon emission tomography, (SPECT)). Invasive monitoring should be restricted to a few when all noninvasive investigations are inconclusive, there is a dual pathology or there is a discordance of noninvasive data. The types of surgery could be curative (resective surgeries: amygdalo hippocampectomy, lesionectomy and multilobar resections; functional surgeries: hemispherotomy) and palliative (multiple subpial transaction, corpus callosotomy, vagal nerve stimulation). Epilepsy surgery in indicated cases has a success range from 50 to 86% in achieving seizure freedom as compared with <5% success rate with AEDs only in persons with DRE. Centers performing surgery should be categorized into Level I and Level II.

It is important to understand that an appropriate selection of a person having drug resistant epilepsy (DRE) is mandatory. This is because presurgical work-up is time- and labor-intensive and has cost implications. All persons of DRE may not be ultimately considered as surgical candidates.

Studies have shown that intractability may be identified quite early at the onset of epilepsy.[1-4]

- Lower efficacy toward the appropriate first AED predicts a high probability of development of DRE.[2,3]
- Ineffective outcome with two appropriate and adequate AEDs is unlikely to achieve seizure freedom with a third drug, the chance being <10%.
- High AED doses do not increase rates of seizure control. They are likely to increase adverse reactions and undesirable side-effects.
- Newer AEDs do not significantly change the above-mentioned equations, and in countries like India, only increase the cost burden.[5-8]
- Failure of response to AEDs should be considered when 2 AEDs tried individually or in combination have failed to control seizures particularly when appropriate AEDs have been used in adequate therapeutic doses (having sufficiently ruled out causes of pseudoresistance like nonepileptic events, syndrome inappropriate medication e.g. JME with CBZ and PHT etc). There is however a trend in trying to identify DRE earlier as this would save the person with DRE several loses, the most important being the quality of life (QOL).

The level of evidences and the grades of recommendations are given in Tables 1 and 2.

**Table 1:** Level of evidence

1.	Systematic review or metaanalysis of randomized controlled trials or at least one randomized controlled trial
2.	At least one well-designed controlled study without randomization or well-designed cohort study
3.	Well-designed nonexperimental descriptive studies, case-control studies and case series
4.	Expert opinion

**Table 2:** Grades of recommendation

A.	Based on Level I evidence
B.	Based on Level II evidence or extrapolated from Level I evidence
C.	Based on Level III evidence or extrapolated from Level I or Level II evidence
D.	Based on Level IV evidence or extrapolated from Level I, Level II or Level III evidence

GPP, good practice point based on the clinical experience of the guidelines developing team

Pediatric epilepsies should be investigated much earlier as uncontrolled seizures during infancy and early childhood are more likely from symptomatic etiologies. Idiopathic pediatric epilepsy patients and cryptogenic epilepsies can expect a respective chance of 95 and 90% of seizure control with AEDs. In contrast, less than 50% children with symptomatic epilepsy can achieve seizure control with AEDs only.[9] The delay in identifying surgical candidates leads to severe irreversible changes in the developing brain, consequently to arrested or delayed development,[10] inducing a "catastrophic epilepsy-induced encephalopathy."[11]

Similarly, disabling seizures should be evaluated for epilepsy surgery much earlier in both adults and children. Typically, seizures that impair consciousness may be called "disabling," whereas simple partial seizures would not be perceived always as being disabling. However, there are no formal criteria for "disabling" or "intolerable" epilepsies as these are subjective categories best decided by the treating neurologist.[5-7]

Likewise, presence of an identifiable surgical substrate like mesial temporal sclerosis, cortical dysplasia, mitotic lesion (not causing any other symptom except epilepsy) should be evaluated early for the possibility of a surgical cure.[1]

In summary, patients are said to have DRE if they have failed two or more AEDs used in their appropriate, adequate dosage, combinations and in appropriate indications after an adequate duration of treatment (not more than 2 years) in adult patients (16 years and above). In pediatric patients, diagnosis of DRE should be made much earlier (sometime even within weeks of onset of seizures), particularly if they present with epileptic encephalopathy, infantile spasms, catastrophic onset of epilepsy, seizure frequency of >1 month and disabling seizures [Table 3].

**Table 3:** Tabular summary for defi nition of DRE, investigative procedures, indications for surgery and guidelines for classifying the level of experise of a center performing epilepsy surgery

Level of evidence	Definition of drug resistant epilepsy (all should be worked-up for epilepsy surgery)
B, C, D	Having failed two AEDs or more, tried on adequate dose: mono followed by rational polytherapy, appropriately for the epilepsy syndrome
Mostly D	Duration of 2 years; more than one seizure per month
	Earlier, if the seizures were "disabling" and prevented the person from having a normal life appropriate for his age and profession
B, C, D	Earlier duration considered for pediatric epilepsy, particularly with epilepsia partialis continua, catastrophic onset, epileptic encephalopathy, disabling seizures, infantile spasms (lesional, e.g. Tuberous sclerosis)
**Investigative procedures**	
B, C, D	Standard
	Interictal EEG: At least three incterictal EEG, both awake and sleep recordings: see guidelines for EEG
	VEEG: At least 3 events if concordant and many more events if discordant/inconclusive.
	MRI: standard sequences: MRI thin slices perpendicular to the hippocampus with at least 1.5 Tesla, closed magnet; T1 and T2 sequences. Special: FLAIR, gradient ECHO, SPGR, MRS, hippocampal volumetry
	Electrocorticography: has been included in standard as inmandatory for neocortical resections
	Special investigations
	Indications: When standard investigations are discordant for substrate-negative pathologies and dual pathologies
	SPECT: Interictal SPECT, ictal SPECT, ictal–interictal subtraction [SISCOS], ictal–interictal subtraction with coregistration on MRI [SISCOM]
	PET: Fdg–PET, other ligands like flumazenil, tryptophan, etc.
	Invasive: depths, grids and strips
**Indications for surgery**	
B, C, D	Surgical substrate with concordance with medical intractability, as defined in I
B, C, D	Substrate negative with pre-electrical (VEEG, EEG), functional imaging (PET, SPECT interictal, ictal SPECT) and intraoperative electrical (invasive VEEG or electrocorticographic) concordance with medical intractability, as defined in I
**Guidelines for an epilepsy surgery center**	
GPP	Level I center:
	Capable of performing "simple*" epilepsy surgeries and emergencies
	1. Electrodiagnostic
	(a) A >24 h VEEG and EEG with surface/sphenoidal recording with supervision by EEG technologist and assistance by epilepsy staff nurse or monitoring technician if necessary
	2. Epilepsy surgery
	(a) Emergency or elective neurosurgery
	(b) Mesial temporal sclerosis
	(c) An established referral agreement with a Level II epilepsy surgical center for surgical procedures for epilepsy, when indicated
	3. Imaging
	(a) MRI with fMRI for language and memory
	4. Pharmacological expertise
	(a) Quality-assured antiepileptic drug levels and 24-h antiepileptic drug level service
	5. Neuropsychological/psychosocial services
	6. Rehabilitation (inpatient and outpatient)
	7. Mandatory expertise
	(a) Neurosurgery
	(b) Neurology
	(c) Internal medicine, pediatrics and general surgery
	*Simple epilepsy surgery: emergency, mesial temporal sclerosis with concordance
	Level II center:
	Capable of performing "complex*" epilepsy surgeries and emergencies
	Includes all capabilities of Level I and, in addition, should be capable of the following:
	1. Electrodiagnostic
	(a) 24-h video/EEG with surface and sphenoidal electrodes
	(b) Invasive VEEG with 24-h recording
	(c) Evoked potential recording
	(d) Electrocorticography
	2. Epilepsy surgery
	Clinical experience of >25 cases per year
	3. Imaging: both standard and special investigations
	4. Team experts
	In addition to those mentioned in Level I, (a) neuroradiologist (b) nuclear medicine specialist (c) psychiatrist
	*Complex epilepsy surgery: includes simple surgeries and all surgeries mentioned in Level II center
**Surgical strategies**	
A, B, C, D	Temporal surgeries
	Anteromedial temporal resection and amygdalohippoacampectomy*,selective amygdalohippocampectomy, lesional resection and lateral temporal resections
	Extratemporal surgeries
	Lesional resections, single-lobe resections, multi-loberesections, hemispherotomy, corpus callosotomy and multiple subpial transaction
	Phase II: grid and depth placement
	Neuromodulatory surgery: vagal nerve stimulation
	Electrocorticography, evoked potentials, neuronavigation andinvasive VEEG required for Level II
	*Level I center, all surgical strategy: Level II

## Rationale for Epilepsy Surgery

Clinical experience and scientific data provide some compelling reasons for considering surgery. Surgical candidates have a much higher chance of attaining surgical freedom as compared with patients receiving medical treatment for DRE (58 vs 8%, respectively).[12] Wiebe *et al*.[12] randomized patients (>16 years) who failed to respond to treatment with two or more anti-epileptic AEDs to medical or surgical arms. Randomization occurred only if they were considered as good surgical candidates. After 1 year of entry into the study, 58% in the surgical group had significant reduction or were seizure free as compared with 8% in the medical group (which had received the best possible medical therapy in lieu of surgery). The percentage of patients in the surgical group who experienced no seizures at all was 38%, vs 3% in the medical group.

Epilepsy itself is associated with a mortality of about 0.5% per year (including all causes, e.g. sudden unexplained death due to epilepsy, from accidents, etc.). Thus, in a person with epilepsy for 2 years, the risk of surgery roughly equals that of mortality from the epilepsy itself (1%).[11] After this, the risk of death from epilepsy becomes more than the surgical treatment.

It has been well demonstrated worldover, including in India,[13-24] that epilepsy surgery performed by trained and experienced hands is safe and is associated with very low mortality and morbidity.

In summary, epilepsy surgery should not be withheld in well-indicated cases as it offers the patient the best chance to get seizure freedom and should be offered early in the course of the disease (and should not be considered as an option of last resort) [Table 3].

## Indications for Epilepsy Surgery

When a patient does not respond to medical treatment and is defined as having drug resistant epilepsy as per the criteria mentioned above, he/she should then be investigated for a possibility of epilepsy surgery. If all the investigations as per the epilepsy surgery protocol (see below) demonstrate a "concordant" epileptogenic foci, the patient would then be a suitable candidate for phase I epilepsy surgery[12-15] [Tables 1‐3].

A well-delineated lesion, like a cavernoma causing epilepsy, is easily picked up on routine magnetic resonance imaging (MRI), but more subtle lesions like mesial temporal sclerosis and focal cortical dysplasias require an epilepsy protocol MRI (at least 1.5 Tesla strength). Not uncommonly several specialized MRI studies performed at different time periods may be required to pick up subtle lesions. It is also very important to perform the MRI with special protocols like Mprage-SPGR, focused surface coils, FLAIR, heavy T2 weighted, MR-spectroscopy and diffusion tensor imaging (DTI) after localization of the epileptogenic zone by video EEG. Thus, it is important that these investigations should be performed at a center having trained specialists (epileptologists, neuroradiologists and neurosurgeons).[25]

It is important to remember that presence of a lesion seen on MRI in a patient with epilepsy does not prove that the lesion is responsible for epilepsy. It is mandatory to perform further investigations like EEG and video EEG to prove concordance with MRI data. Not uncommonly, there are no lesions seen even on specialized MRI studies and the presence of an "epileptogenic zone" may be validated by advanced investigations like ictal SPECT (with SISCOS/SISCOM), PET, video electroencephalography (VEEG) and invasive VEEG (phase II surgery). Such a zone would be classified under "substrate negative."

**Figure 1:**
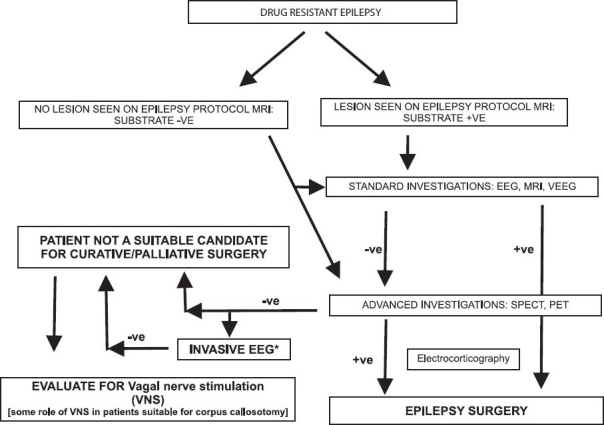
Flow chart showing a management paradigm for patients being subjected to epilepsy surgery

In summary, presence of a "concordant" epileptogenic focus in a patient with DRE would form an indication for "resective" epilepsy surgery. If such a focus is not detected, the patient may still be evaluated by advanced techniques and phase II surgery. Patients who have syndromes like Lenox Gestaut syndrome or intractable disabling seizures without delineation of an epileptogenic zone may be candidates for "palliative" surgery, such as corpus callosotomy, multiple subpial transaction or vagal nerve stimulation. *Absence of a lesion even on specialized MR images does not exclude that the person will not benefit from epilepsy surgery* [Figure 1].

## Investigations for Epilepsy Surgery

Before surgery, a careful presurgical evaluation is mandatory. The purpose is to delineate the epileptogenic zone, defined as "the area of cortex indispensable to the generation of epileptic seizures." It is important to establish its relationship to eloquent cortex as the surgery should not result in a new deficit.[26] At present, the epileptogenic zone cannot be localized directly by any single diagnostic test.[1,16,26] The minimum investigations required are specialized MRI sequences, video EEG, documenting a minimum of three concordant and habitual seizures. In cases where the MRI and video EEG are discordant or there exists a dual pathology, or MRI is negative, advanced investigations like ictal SPECT (with SISCOS/SISCOM), PET and invasive video EEG are required. Centers that are equipped with a functional MRI need to perform language, memory and motor functional delineation when the epileptogenic zone is close to the eloquent areas. Magnetoencephalography is an important tool for source localization, but is currently unavailable in the country.[1,2,25,27,29,30]

The importance of neuroradiology expertise and optimal MRI is demonstrated by a study where (*n* =123) standard MRI interpreted by nonepilepsy-trained neuroradiologists revealed focal lesions in only 39% of the cases, whereas the images analyzed by epilepsy-trained neuroradiologists showed focal abnormalities in 91%. With respect to prediction of a neuropathologic diagnosis, only 22% of cases imaged via standard MR and analyzed by nonepilepsy-specialist radiologists were correct, as opposed to 89% imaged via epilepsy-protocol MR and interpreted by epilepsy-specialist neuroradiologists.[25]

Noninvasive studies are usually sufficient for presurgical evaluation in a majority of epilepsy patients (e.g. 70–80% of mesial temporal lobe epilepsy patients). However, if this is still discordant, patients may undergo invasive tests such as ictal EEG recordings via intracranial depth or subdural strip and grid electrodes.

In summary, the localization of the "epileptogenic zone" cannot be performed by any single investigation. It has to be localized by multiple investigations, which are of four broad categories: (1) structural imaging: MRI as per the epilepsy protocol, (2) electrical localization: EEG, VEEG, (3) functional imaging: PET, SPECT, fMRI. [Wada test (an invasive test) is being replaced by fMRI.], (4) the need for advanced investigations should be taken only after the epilepsy surgery team meets and formulates a hypothesis about the probable epileptogenic zone as these tests are cost- and labor-intensive [Table 3].

The investigations are tabulated below:

### Standard investigations

Interictal EEG: At least three incterictal EEGs, both awake and sleep recordings: see guidelines for EEG.

VEEG: Should be performed at a centre equipped to perform this with trained epileptologists, nursing staff and technicians to interpret the results and take care of safety and quality issues. Ideally should be reported and seen by at least 2 epileptologists, trained and certified for the same.

MRI: Standard sequences: high-resolution MRI (>1.5 Tesla) with phase-arrayed surface coils to improve the signal-to-noise ratio are required. High-resolution MR scans should be obtained with a dedicated epilepsy-protocol: Thin 3-mm slices perpendicular to the hippocampus (slices are angulated along the long axis of the hippocampus) with T2-weighted and FLAIR sequences that are sensitive to hippocampal sclerosis, while 3D T1-weighted gradient-echo and inversion recovery sequences aid in the detection of cortical dysplasias. If primary motor cortex, primary sensory cortex or dorsal frontal lobe lesions are suspected, angulation is along the anterior commissure-posterior commissure line. When the acquired images raise suspicion for potentially enhancing lesions, such as tumors or vascular malformations, additional axial and coronal T1-weighted spin-echo sequences are obtained before and after gadolinium injection. MpRage/SPGR will also need to be performed, especially after the focus is ascertained by VEEG.

### Advanced investigations

Indications: When standard investigations are discordant for substrate-negative pathologies and dual pathologies.

SPECT: Interictal SPECT, ictal SPECT, ictal–interictal subtraction (SISCOS), ictal–interictal subtraction with coregistration on MRI (SISCOM).

PET: Fdg-PET, other ligands like flumazenil/tryptophan under investigation.

Invasive: (Depths, grids and strips) Here, the electrodes are implanted either through a stereotactic frame or by an open craniotomy. The following are examples of instances that may require invasive intracranial monitoring:[28]

1. Seizures are lateralized but not localized (e.g., a left-sided, widespread frontal–temporal onset). Seizures are localized but not lateralized (e.g., ictal EEG patterns that appear maximally over both temporal lobes).
2. Seizures are neither localized nor lateralized (e.g., stereotyped complex partial seizures with diffuse ictal changes or initial changes obscured by artifact).
3. Seizure localization is discordant with other data [e.g., EEG ictal scalp data discordant with neuroimaging (MRI, PET, SPECT) or neuropsychological data].
4. Relationship of seizure onset to functional tissue must be determined (e.g., seizures with early involvement of language or motor function).
5. Relationship of seizure onset to lesion must be determined (e.g., dual pathology or multiple intracranial lesions).
6. If seizures are clinically suspected but video-EEG is inadequate for defining them [e.g., simple partial seizures with no detectable scalp EEG ictal discharge or suspected epileptic seizures with unusual semiology that suggests psychogenic seizures (pseudo-pseudo seizures)].

In India, particularly with financial restraints, it becomes very important to tailor the investigations carefully. All the above investigations are not required in all the cases, i.e., where the importance of the team approach comes in and an epilepsy surgery case conference should be done. Wada's test cannot also be performed in all centers due to the commercial availability of sodium amytal. It is more commonly being replaced with fMRI for language and memory.

### Types of surgical interventions

The surgical interventions may be broadly divided into (a) temporal and (b) extratemporal surgeries. From an outcome point of view, a more useful classification would be (a) definitive: offering complete surgical cure or at least 90% chances of reduction in seizures and (b) palliative: offering reduction of the seizure load rather than cure. From a strategy point of view, the latter classification would be a more rational approach. The evidence till date is briefly stated.[10,12-14,28]

#### Temporal surgeries

Approximately two-thirds of the patients become free of seizures, excepting simple partial seizures, (aura only) after anterior temporal lobectomy. This outcome was found in a large number of Class IV series and was confirmed in a randomized, controlled trial of surgery vs anti-epileptic drug therapy. Ten to 15% do not alter after surgery.[12,13]

QOL scores improve after temporal lobectomy, but clinical significance of these measures have not been studied.[14,18,21] Psychiatric outcome and neuropsychologic and psychosocial function after surgery usually improves, with worsening related predominantly to the persistence of seizures. Employment status and activities of daily living in general improve, mortality is decreased and medication regimens are reduced about 1 year after surgery gradually.[18] Stopping the drugs is to be done only in select cases as chance of recurrence is dependent on several factors. Hence the patient usually remains on a moderate dose of at least one drug 2-3 years after surgery, this decision should be taken only at a trained centre preferably by the epileptologist.

#### Extratemporal surgeries

About 45–70% of patients with extratemporal foci become seizure-free after surgery. This is however an average figure and the outcome depends on the type of surgery. For e.g., the seizure freedom attained in hemisphertomy is quite high (75–85%) while it is quite low (<30%) for procedures like multiple subpial transactions and corpus callosotomy.

A complete description is beyond the scope of the proposed guidelines. The type of surgeries provided below only attempt to provide an idea of all the spectra available in epilepsy surgery. The ultimate decision depends on the surgeon after due discussion with the epilepsy surgery team [Table 3].

Anteromedial temporal lobectomy with amygdalo-hippocampectomy: A surgical procedure where the anterior and the medial part of the temporal lobe resected along with hippocampus, amydala, uncus and the mesial structures. This is mostly indicated for epilepsies arising from the medial temporal lobe.

Selective amygdalo-hippocampectomy: A more technically demanding surgical procedure where only the mesial structures, like hippocampus, amydala and uncus, are removed, leaving the lateral temporal lobe intact. Its role over the earlier described procedure is not certain.

Multiple subpial transection: A surgical procedure coming under the category of "palliative" procedure where the aim is to reduce the seizure burden only rather than to eliminate them completely. It is usually performed on an eloquent cortex like the mortor cortex so as to avoid producing any deficit. Here, the gyrus is divided into small blocks of 1 × 1 cm using a special instrument.

Hemispherotomy: A complex surgical procedure where the entire affected hemisphere (in conditions like Rasmussen's syndrome) is disconnected from the opposite hemisphere. This is much less invasive than the procedure, like the earlier hemispherectomy (where the hemisphere is disconnected and then physically removed). The latter procedure has now been given up due to the higher incidence of complications, like blood loss, hemosiderosis, etc.

Electrocorticography: An investigation to determine when different sizes of electrodes (strips, grids) should be placed on the surface of the brain to localize the "epileptogenic" focus before resection in all patients' neocortical temporal and extra-temporal locations with concordant investigations. It is also to be used in tailored resections in hippocampal sclerosis[31,32].

## Postsurgery AED Management

It is important that all AEDs be continued after surgery. Tapering of medicine must be carried out in consultation with the team performing the surgery. This must be done after adequately looking into the pathology and postoperative EEGs.

## Conclusion

Epilepsy surgery must NOT be a LAST RESORT as a treatment option. Any patient being treated by a general physician/pediatrician not responding to medication and continuing to have seizures impairing the QOL must be referred to a trained epileptologist and center having capability in investigating patients and assisting in determining the cause of intractability and hence a possible surgical cure.[33]
